# Supplementary material for: Electric control of optically-induced magnetization dynamics in a van der Waals ferromagnetic semiconductor
Source: Nat Commun. 2024 Feb 12;15:1298. doi: 10.1038/s41467-024-45623-2 (PMC10861592; doi:10.1038/s41467-024-45623-2)
Supplement: Supplementary file 1 — Supplementary Information [file 41467_2024_45623_MOESM1_ESM.pdf]

Supplementary Information for 'Electric control of optically-induced magnetization dynamics in a van der Waals ferromagnetic semiconductor'

1 Atomic force microscopy

The thicknesses of the flakes of the sample were measured using atomic force microscopy (AFM). The height map is shown in Fig. 1a. The thickness of the hexagonal boron nitride (hBN) flakes are determined from the line trace along the blue line, shown in Fig. 1b, yielding 20 nm for the bottom and 21 nm for the top flake respectively. The $Cr_2Ge_2Te_6$ (CGT) thickness was extracted from the line profile along the green line, shown in Fig. 1c, yielding 10 nm.

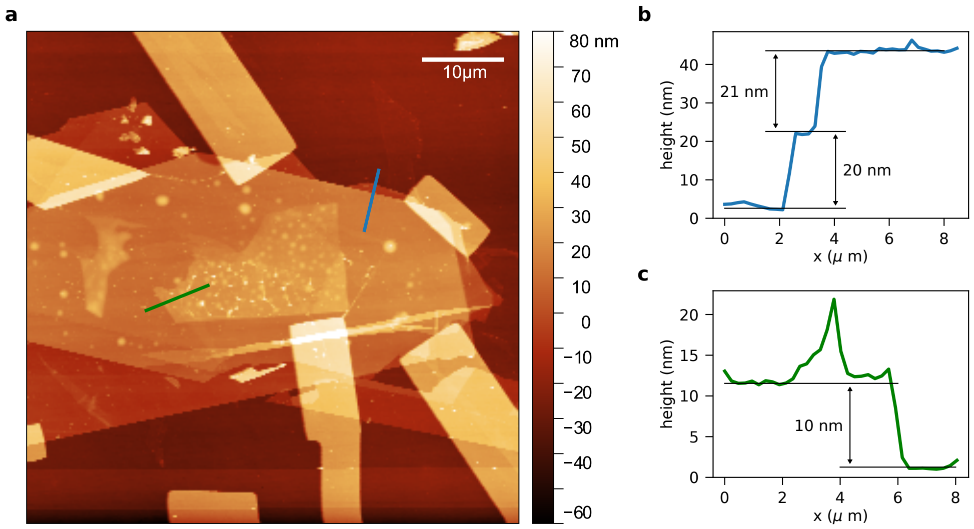

Fig. 1 Atomic force microscopy scan. a, AFM height image. Height profiles taken along the blue and green line are used to extract the thickness of the top and bottom hBN, and of the thickness of the CGT respectively. **b**, Height profile taken along the blue line to extract the thickness of the top (21 nm) and bottom (20 nm) hBN flakes. **c**, Height profile taken along the green line to extract the thickness of the CGT flake (10 nm).

2 Finding optimal parameters for the Faraday effect

The sensitivity of the Faraday effect on changes in the magnetization depends on the wavelength. Additionally, the change in rotation and ellipticity of the light caused by the Faraday effect are generally different. To find the optimal wavelength and polarization mode, we probe the magnetization using the Faraday effect while sweeping the magnetic field. The change in polarization when the magnetic field reverses the magnetization direction of the CGT indicates the sensitivity of the Faraday effect. We determine this sensitivity for various values of the wavelength, ranging from 830 nm to 940 nm, measuring change in rotation and ellipticity simultaneously. We found that the ellipticity at 870 nm had the highest sensitivity, and therefore we use this wavelength and polarization mode for the time-resolved Faraday ellipticity (TRFE) measurements.

3 Gate voltage dependence of magnetization curves measured by static Faraday ellipticity

We measured the gate dependence of the magnetization curves of CGT by means of the Faraday effect using a pulsed laser with a fluence of 5.7 μJ/cm^2 at a wavelength of 870 nm. We measured the change in ellipticity of the light, as described in the Methods. The gate dependence of the magnetization curves for various values of Δn and ΔD are presented in Fig. 2.

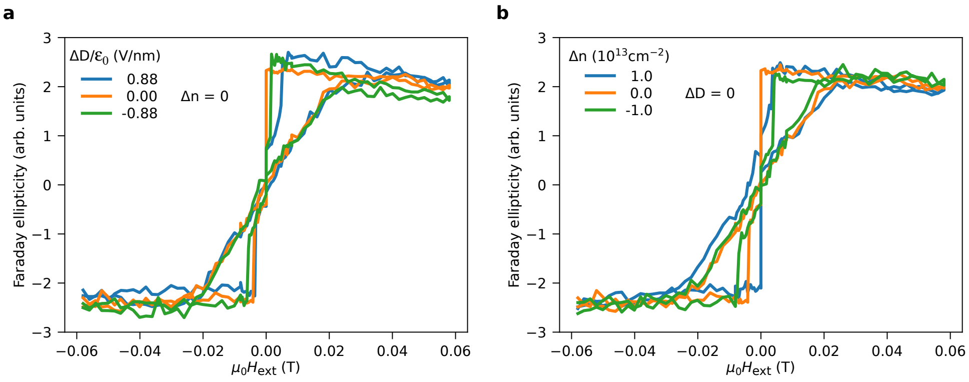

Fig. 2 Gate dependence of the magnetization curves measured by static Faraday ellipticity. a, Effect of ΔD on the magnetization curves for $\Delta n = 0$. **b**, Effect of Δn on the magnetization curves for $\Delta D = 0$.

In addition, we measured the magnetization curves at 920 nm using the polar magneto-optic Kerr effect (MOKE) in a different experimental setup as a function of Δn at $\Delta D = 0$. From this data we extracted how the coercive field changes with Δn, which is shown in Fig. 3, to check if it is similar to the Δn dependence of

H_{int}. We extract the coercive field by individually fitting the trace and retrace of the magnetization curves with the formulas

$$y_{\text{trace}} = \begin{cases} A \tanh\left(\frac{x - x_{0,t}}{w}\right) + ax + b, & \text{if } x > x_{\text{s,t}} \\ A + ax + b, & \text{otherwise} \end{cases} \tag{1}$$

$$y_{\text{retrace}} = \begin{cases} A \tanh\left(\frac{x - x_{0,r}}{w}\right) + ax + b, & \text{if } x < x_{\text{s,r}} \\ A + ax + b, & \text{otherwise} \end{cases} \tag{2}$$

where A is half of the difference in MOKE ellipticity for large positive and negative fields, x_0 the horizontal shift, w the width of the tanh (which is proportional to the saturation field), and x_s the position of the sharp switch close to zero field that indicates the transition from a single domain to multi domain state [1]. The parameters a and b are respectively the slope and offset of the background. The coercive field is generally not fitted well. To get a better estimate for the coercive field, we subtract the background, and separately fit a straight line, with equal slope, through the trace and retrace where the signal is close to zero and approximately linear (we used the signal value < 0.002). The coercive field is then calculated as half of the horizontal separation of these two lines. The graph of $\mu_0 H_c$ versus Δn presented in Fig. 3 is very similar to the graph of $\mu_0 H_{\text{int}}$ versus Δn at $\Delta D = 0$ presented in Fig. 2d in the main text. This means that the Δn dependence of H_{int} obtained from TRFE measurements of the magnetization precession is consistent with the Δn dependence of H_c obtained from static MOKE measurements.

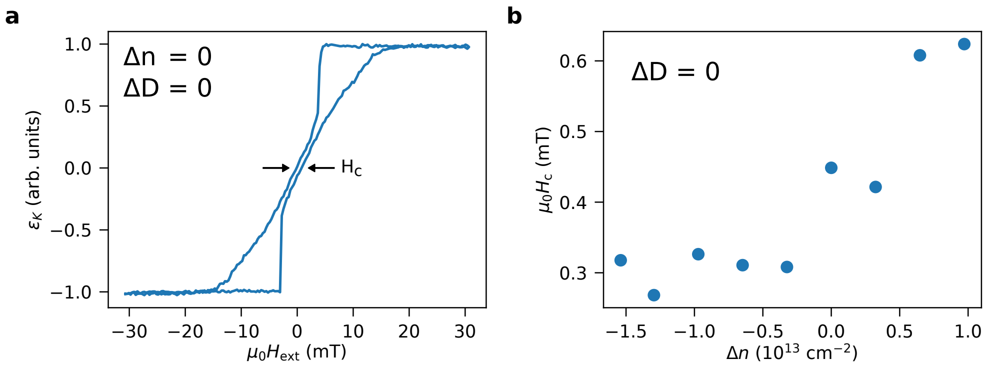

Fig. 3 Coercive field from static MOKE measurements a, Magnetization curve measured by static MOKE at $\Delta n = \Delta D = 0$. The coercive field is half of the horizontal shift of the trace and retrace near zero. **b**, Effect of Δn on the coercive field at $\Delta D = 0$.

4 Equation for ferromagnetic resonance

We use Eq. (1) of the main text to describe the ferromagnetic resonance (FMR) frequency of our CGT sample. In the literature the expression for the ferromagnetic resonance frequency is usually written in a different manner, which is less compact. This section shows that our expression is equivalent to the more common expressions found in literature, which is usually of the form [2].

$$
\begin{aligned}
f &= \sqrt{f_0^2 + 1/(2\pi\tau)^2} \\
f_0 &= \frac{\gamma\mu_0}{2\pi}\sqrt{H_1 H_2}/\sqrt{1+\alpha^2} \\
1/\tau &= \alpha\gamma\mu_0\left(H_1 + H_2\right)/(1+\alpha^2) \\
H_1 &= H_{\text{ext}}\cos(\theta_H - \theta_M) + H_{int}\cos^2(\theta_M) \\
H_2 &= H_{\text{ext}}\cos(\theta_H - \theta_M) + H_{int}\cos(2\theta_M),
\end{aligned}
$$

In these equations, f is the FMR frequency, τ the decay time of the oscillation, γ the gyromagnetic ratio, α the Gilbert damping, H_{ext} the external magnetic field, H_{int} the effective internal field due to magnetocrystalline anisotropy and demagnetizing field, θ_M the angle between the magnetization vector and the sample normal, and θ_H the angle between the external magnetic field and the sample normal. Note that unlike in Ref. [2], these equations are in SI units. When damping is small, α^2 can be neglected. The expression for f now simplifies to

$$
\begin{aligned}
f = f_0 &= \frac{\gamma\mu_0}{2\pi}\sqrt{H_1 H_2} && (3) \\
H_1 &= H\cos(\theta_H - \theta_M) + H_{\text{int}}\cos^2(\theta_M) && (4) \\
H_2 &= H\cos(\theta_H - \theta_M) + H_{\text{int}}\cos(2\theta_M) && (5)
\end{aligned}
$$

Next, we identify H_1 as the strength of the total effective magnetic field, $\mathbf{H}_{\text{eff}} = \mathbf{H}_{\text{ext}} + H_{\text{int}}\cos(\theta_M)\hat{\mathbf{n}}$, where $\hat{\mathbf{n}}$ is the sample normal unit vector. Key to this derivation is the observation that in equilibrium, $\mathbf{H}_{\text{eff}}$ and $\mathbf{M}$ are parallel, meaning that $\mathbf{H}_{\text{eff}}/|\mathbf{H}_{\text{eff}}| = \mathbf{M}/|\mathbf{M}|$

$$
\begin{aligned}
|\mathbf{H}_{\text{eff}}| &= \frac{|\mathbf{H}_{\text{eff}}|^2}{|\mathbf{H}_{\text{eff}}|} = \mathbf{H}_{\text{eff}}\cdot\frac{\mathbf{H}_{\text{eff}}}{|\mathbf{H}_{\text{eff}}|} \\
&= \mathbf{H}_{\text{eff}}\cdot\frac{\mathbf{M}}{|\mathbf{M}|} \\
&= (\mathbf{H}_{\text{ext}} + H_{\text{int}}\cos(\theta_M)\hat{\mathbf{n}})\cdot\frac{\mathbf{M}}{|\mathbf{M}|} \\
&= |\mathbf{H}_{\text{ext}}||\mathbf{M}|\cos(\theta_H - \theta_M)/|\mathbf{M}| + H_{\text{int}}\cos(\theta_M)|\hat{\mathbf{n}}||\mathbf{M}|\cos(\theta_M)/|\mathbf{M}| \\
&= H_{\text{ext}}\cos(\theta_H - \theta_M) + H_{\text{int}}\cos^2(\theta_M) \\
&= H_1 && (6)
\end{aligned}
$$

To arrive at the final expression of $|\mathbf{H}_{\text{eff}}| = H_1$, we have used the property of the inner product that $\mathbf{v}_1 \cdot \mathbf{v}_2 = |\mathbf{v}_1||\mathbf{v}_2|\cos(\theta_{\mathbf{v}1,\mathbf{v}2})$. To show that $H_2 = |\mathbf{H}_{\text{eff}}| - H_{\text{int}} \sin^2 \theta_M$, we need the trigonometric identities $\cos(2\theta_M) = (2\cos^2(\theta_M) - 1)$ and $\cos^2(\theta_M) + \sin^2(\theta_M) = 1$.

$$\begin{aligned} H_2 &= H_1 - H_{\text{int}} \cos^2(\theta_M) + H_{\text{int}} \cos(2\theta_M) \\ &= H_1 - H_{\text{int}} \left(\cos^2(\theta_M) - \cos(2\theta_M)\right) \\ &= H_1 - H_{\text{int}} \left(\cos^2(\theta_M) - 2\cos^2(\theta_M) + 1\right) \\ &= H_1 - H_{\text{int}} \left(1 - \cos^2(\theta_M)\right) \\ &= |\mathbf{H}_{\text{eff}}| - H_{\text{int}} \sin^2(\theta_M) \end{aligned} \tag{7}$$

Combining Eq. (3) with (6) and (7), and using $\gamma = g\mu_B/\hbar$, gives

$$f = \frac{g\mu_B\mu_0}{2\pi\hbar} \sqrt{|\mathbf{H}_{\text{eff}}| \left(|\mathbf{H}_{\text{eff}}| - H_{\text{int}} \sin^2(\theta_M)\right)}. \tag{8}$$

This is exactly Eq. (1) of the main text. Therefore, our expression for the FMR frequency is equivalent to the expression found in e.g. Ref. [2].

5 θ_H dependence of g and H_{int}

We extract the g-factor and the internal effective field (H_{int}) from the external magnetic field (H_{ext}) dependence of the magnetization precession frequency (f), as explained in the main text and the Methods. To obtain g and H_{int}, we need to fix the value of the angle θ_H between H_{ext} and the sample normal. Fig. 4 shows the gate dependence of g and H_{int} for different values of θ_{H}, ranging from 45 degrees to 60 degrees. Panels a, d, g, and j show that for $\theta_{\text{H}} = 45°$ and 50° the data is fitted best. Since 50° is more likely based on how we mounted the sample in the cryostat, we have chosen $\theta_{\text{H}} = 50°$ for our analysis, despite the observation that $\theta_H = 60°$ results in a g-factor that is more in line with values reported in literature. From the results presented in Fig. 4, we can conclude that, apart from an offset of the mean value, the gate dependence of g and H_{int} hardly changes with θ_n, even if we choose θ_{H} such that we obtain a g factor that agrees with literature values. Therefore, the conclusions in the main on the gate dependence of the H_{int} do not depend on the exact value of θ_H, as long as these values are reasonable.

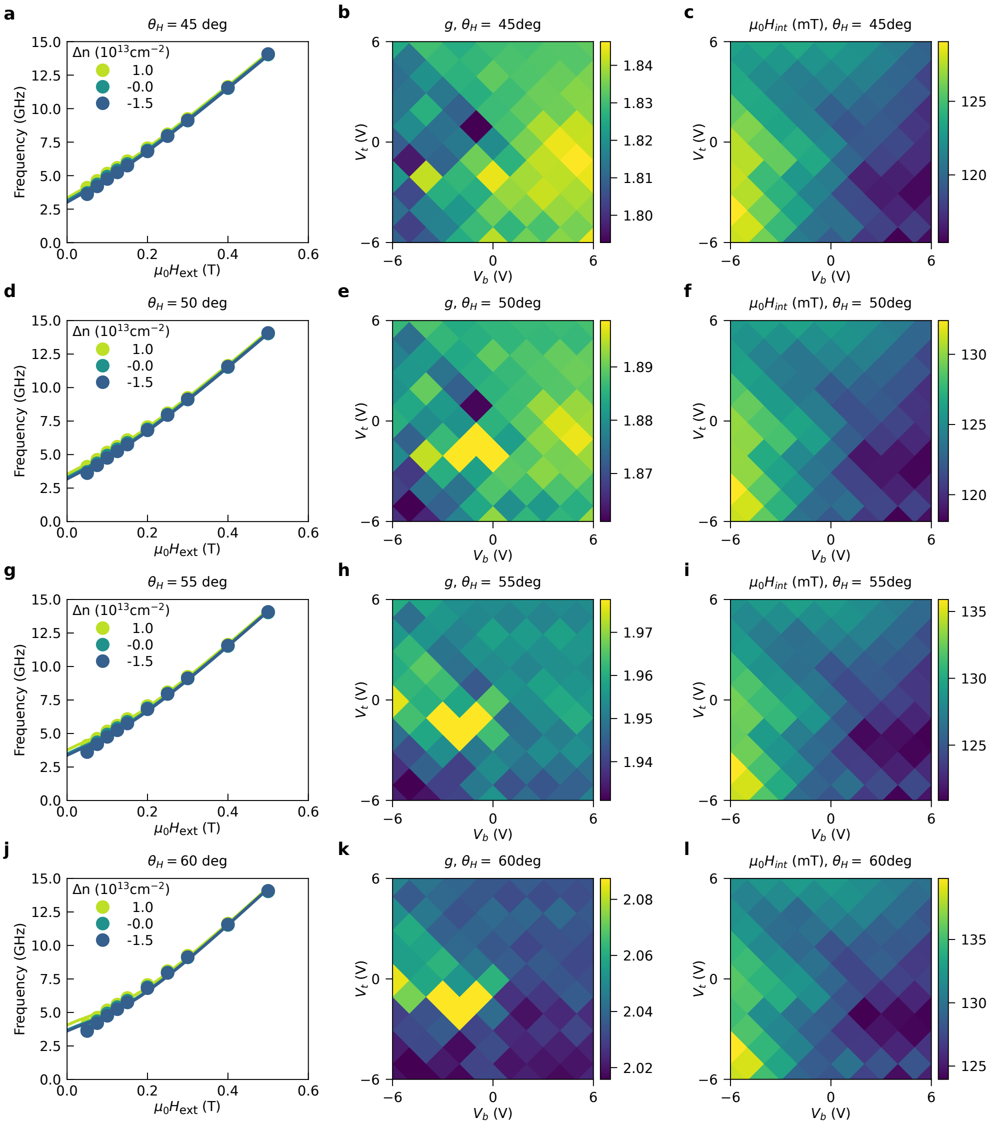

Fig. 4 Effect of the sample mounting angle on g and H_{int}. Fit of precession frequency versus H_{ext} (**a**) data to obtain g (**b**) and H_{int} (**c** for) $\theta_{\mathrm{H}} = 45$ deg. Results for $\theta_H = 50, 55$ and 60 degrees are presented in respectively **d-f**, **g-h** and **j-l**.

6 Pump fluence dependence of magnetization precession

We found that the frequency and amplitude of the magnetization precession depends on the fluence of the pump pulse, while the starting phase is largely unaffected. For the data presented in the main text, the pump fluence was measured to be 25 μJ/cm^2. Fig. 5 shows how the TRFE signal is affected by different values of the pump fluence. The frequency, amplitude and starting phase of the oscillation for each of these measurements is extracted by fitting the data with Eq. (2) of the Methods, and is plotted in respectively Fig. 6a, b and c.

In Fig. 6a it can be seen than the magnetization precession frequency decreases with pump fluence for all the extreme gate voltage combinations. The precise rate at which the frequency depends on the pump power depends on the applied gate voltages. This can be due to the photo-induced magnetic anisotropy effect (PIMA) since an increase in pump fluence would result in an increase (or decrease) of the population of the dopant or impurity states. Since the PIMA effect depends on the population of these states, an increase in the pump fluence should result in an increase of the effective field generated by it. The change in the observed frequency should then depend on the specific sign (with respect to the magnetic anisotropy and applied magnetic field) of the PIMA. Additionally, a decrease in the saturation magnetization (M_s) and magnetocrystalline anisotropy caused by heating by the laser could offer another possible explanation. This does affect the values of the Landé g-factor and the effective internal field (H_{ext}). Since we used the same pump fluence for all measurements, it does not affect the general trends we observe for the gate voltage dependence of the magnetization precession frequency, starting phase, and amplitude shown in this work.

Fig. 6b shows that the amplitude of the TRFE oscillations increases with increasing pump fluence, up to a fluence of about 35 μJ/cm^2, where the graph seems to level off. For low pump fluences, the amplitude of the magnetization precession is expected to increase linearly. This is because all magnetization dynamics inducing effects that we consider (ΔK mechanism, ICME, and PiMA) are linear in the pump fluence for small fluences. The deviation from this linear trend that is visible in Fig. 6b can have several explanations. One possible explanation is that the magnetization dynamics inducing effects start to become nonlinear with pump fluence. For example, this could be caused by saturation of the laser pulse absorption, or by photodoping that changes the strengths of the mechanisms at play. Another possible explanation is that the magnitude of the magnetization starts to decrease significantly due to laser heating, and thereby reducing the TRFE signal . Since Fig. 6c shows that the starting phase of the oscillations is practically independent of the laser fluence, the ratio of the strength of the dominant magnetization dynamics inducing mechanisms does not change. Therefore, a decrease in magnetization is likely to be the most important reason why the TRFE oscillation amplitude is not linear with the pump fluence.

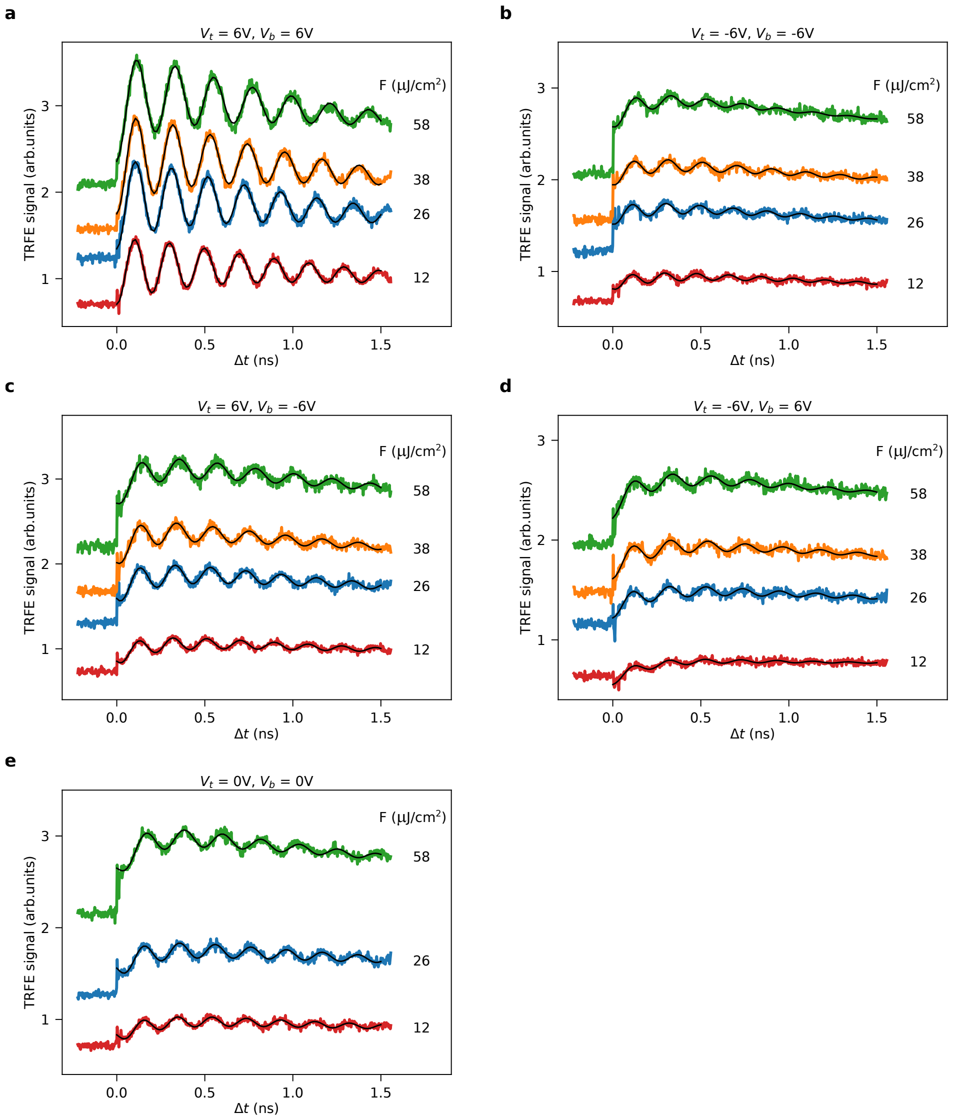

Fig. 5 Pump fluence dependence of the TRFE measurements. Dependence of the TRFE signal on the pump fluence, measured at $\mu_0 H_{\text{ext}} = 100$ mT, for various values of V_t and V_b. The solid lines indicate the best fit of Eq. (2) in the Methods, which is used to extract the magnetization precession frequency.

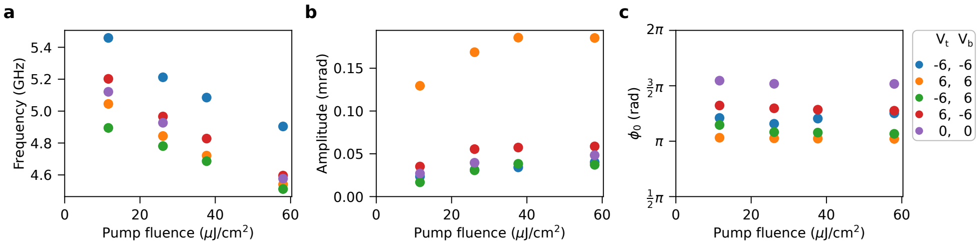

Fig. 6 Pump fluence dependence of the TRFE oscillations. a, b, c, Dependence of the precession frequency (a), amplitude (b) and starting phase (c) on the pump fluence, measured at $\mu_0 H_{\text{ext}} = 100$ mT, extracted from Fig. 5. All the extreme gate voltage combinations used for the gate voltage scans of the magnetization dynamics (Figs. 2a, 3a, and 3b of the main text) are shown.

7 Photodoping

The number of photo-induced charges by the pump laser can be estimated as follows. The pump laser has an average output power of 400 μW at a repetition rate of 80 MHz. The energy per pulse is then $400\,\mu\text{W}/(80\,\text{MHz}) = 5.0\times10^{-12}J$. Since the photon energy of the pump at 870 nm is $E_{\text{photon}} = hc/\lambda = 2.29\times10^{-19}$J, each pulse consists of $E_{\text{pulse}}/E_{\text{photon}} = 5.0\times10^{-12}\,\text{J}/(2.29\times10^{-19}\,\text{J}) = 2.2\times10^{7}$ photons. The pump pulse is focused to a 9 μm^2 spot (diameter is full width at half maximum of intensity profile). However, since the pump hits the sample at an angle of 50°, the spot gets elongated by a factor of $1/\cos(50°) \approx 1.55$, resulting in $1.4\times10^{7}/(9\times10^{-8}) = 1.6\times10^{14}$ photons per square centimeter.

To estimate the fraction that gets absorbed by the CGT, we use the values of the optical conductivity tensor σ of bulk CGT calculated by Fang et. al. [3]. For λ = 870 nm this is about $(1.2-2i)10^{15}$ Hz (Gaussian units). The refractive index can be calculated from the optical conductivity via $n = \sqrt{1+4\pi i\sigma/\omega} = \sqrt{1+2i\sigma\lambda/c} = 3.8+0.95i$. The intensity of light as a function of distance d traveled through the CGT is $I = I_0 e^{-4\pi d/\lambda} = I_0 e^{-0.014d/\text{nm}}$. The absorbed fraction of the intensity for 10 nm of CGT is $1 - I(10\text{nm})/I_0 = 0.14$. Therefore, about 14% of the photons that travel through the CGT get absorbed. Since a part of the photons that arrive at the CGT gets reflected, we take the fraction of photons arriving at the CGT that get absorbed to be in the order of 10%. This results in $0.1\times1.6\times10^{14}\,\text{cm}^{-2} = 1.6\times10^{13}$ photons that create electron-hole pairs. Therefore, the photo-induced charge carrier density right after excitation of the pump pulse is of the order of the maximum gate-induced charge carrier density.

We would like to stress that even though the photo-induced carrier concentration is of the order of the gate-induced carrier concentration, the energy profile of the two are drastically different. While electrostatic doping changes the overall doping level (background), laser excitation generates high energy electrons and holes, which rapidly decay and thermalize towards lower energies. Since we use ultrashort laser pulses and not continuous wave lasers, the photo-induced carrier density changes over time. Additionally, we would like to point out that while the carrier excitation densities are indeed comparable to the electrostatic doping, we still observe drastic gating effects on the magnetization dynamics. Finally, our TRFE measurements show a very similar response to electrostatic gating for different pump fluences, as is shown in Supplementary Fig. 5. Therefore we are confident that the photo-induced carrier density does not affect our main findings on the effect of gating on the magnetization dynamics properties, and on the mechanisms of optically induced magnetization dynamics in CGT.

8 Damping of magnetization precession

The damping time of the magnetization precession (τ_{osc}) is an important parameter to describe the magnetization dynamics. As mentioned in the main text, the precession frequency we measured is well described by the Landau-Lifshitz-Gilbert (LLG) equation in the limit of low damping. In the LLG equation, the effective damping is included through the phenomenological dimensionless parameter α_{eff}.

This damping describes how the magnetization relaxes to its equilibrium state after excitation. The effective damping parameter α_{eff} can be decomposed into a frequency-independent intrinsic damping α, and a frequency-dependent extrinsic damping α_{ext}, resulting in $\alpha_{\text{eff}} = \alpha + \alpha_{\text{ext}}$. For our measurements we assume that the main source of extrinsic damping is spatial fluctuations in H_{int}. This contributes to the damping via inhomogeneous broadening of the precession frequency, and is quantified by $\alpha_{\text{ext}} = \sqrt{2\ln(2)}\,|\mathrm{d}\omega/dH_{\text{int}}|\,\Delta H_{\text{int}}/\left[\frac{g\mu_B\mu_0}{\hbar}(2\,|\mathbf{H}_{\text{ext}}| - H_{\text{int}}\sin^2(\theta_M))\right]$ [4]. Here, g is the Landé g-factor, μ_B the Bohr magneton, μ_0 the vacuum permeability, $\mathbf{H}_{\text{ext}}$ the external magnetic field, θ_M the angle between the magnetization and the sample normal, ω the angular frequency of the precession, and $H_{\text{int}} = 2K_u/(\mu_0 M_{\text{s}}) - M_{\text{s}}$ with K_u the uniaxial magnetic anisotropy energy and M_{s} the saturation magnetization. Note that in [2] $|\mathbf{H}_{\text{eff}}|$ is expressed as $H_{\text{ext}}\cos(\theta - \theta_M) + H_{\text{int}}\cos^2(\theta_M)$. The angle θ_M is calculated by minimizing the magnetic energy in the presence of an external field, perpendicular magnetic anisotropy, and shape anisotropy [4]. The derivative $|\mathrm{d}\omega/dH_{\text{int}}|$ is calculated numerically using Eq. (1) of the main text. For large external magnetic fields, ΔH_{int} becomes less relevant, and $\alpha_{\text{eff}} \approx \alpha$.

The expression for the magnetization precession damping time (τ_{osc}) obtained from the ferromagnetic resonance (FMR) mode of the LLG equation for small values of α_{eff} is given by [2, 4]

$$\tau_{\text{osc}} = 2\hbar/\left[g\mu_B\mu_0\alpha_{\text{eff}}\left(2\,|\mathbf{H}_{\text{ext}}| - H_{\text{int}}\sin^2(\theta_M)\right)\right]. \tag{9}$$

To obtain the damping parameters α and ΔH_{int}, this equation is fitted to the values of τ_{osc} extracted from the TRFE measurements at various values of Δn with $\Delta D = 0$, which is presented in Fig. 7. We used the values $g = 1.886$ and $\mu_0 H_{\text{int}} = 134$, 125 and 120 mT for respectively $\Delta n = 1.0$, 0 and $-1.5 \times 10^{13}\text{cm}^{-2}$, which are obtained from fitting the precession frequency versus H_{ext} data using Eq. (1) of the main text. The fit results for the damping parameters are summarized in Tab. 1. As can be seen in Fig. 7, the data for $\Delta n = -1.5 \times 10^{13}\text{cm}^{-2}$ is fitted well by Eq. (9). The data for $\Delta n = 1.0 \times 10^{13}\text{cm}^{-2}$ is fitted reasonably well, but for $\Delta n = 0$ the fit is quite poor, even when taking the errors in τ_{osc} into account.

A bad fit likely results from the large spread of the measurement points at high external magnetic fields, and (when present) from the peak in τ_{osc} around $H_{\text{ext}} = 200$ mT. This peak, which starts around $H_{\text{int}} \approx 125$ mT, consistently shows up for other small values of Δn and ΔD as well. A possible explanation for this is that there are more factors affecting the extrinsic damping than ΔH_{int} alone. The large spread and errors in τ_{osc} for $\Delta n = 0$ and $1.0 \times 10^{13}\text{cm}^{-2}$ are caused by a combination of two factors. The first factor is the low precession amplitude, which results in a lower signal to noise ratio for all fitting parameters related to the amplitude of the precession. This can be improved by inducing precession with a larger amplitude (e.g. higher pump fluence), or by reducing the noise in the system (e.g. higher probe fluence, or better blocking of pump before the detection stage). The second factor is the relatively short maximum pump-probe delay (Δt_{max}) that we can achieve in our experimental setup, which is about 1.5 ns. For a good estimate of the magnetization precession damping time, $\Delta t_{\text{max}} \gg \tau_{\text{osc}}$. In our case however, $\Delta t_{\text{max}} \approx \tau_{\text{osc}}$.

Using all three fit results, we obtain an intrinsic Gilbert damping of $\alpha \approx 6 \times 10^{-3}$, and a spread in the internal effective field of $\Delta H_{\text{ext}} \approx 9$ mT. Both of these values are in line with values found in literature for thin CGT flakes [4, 5].

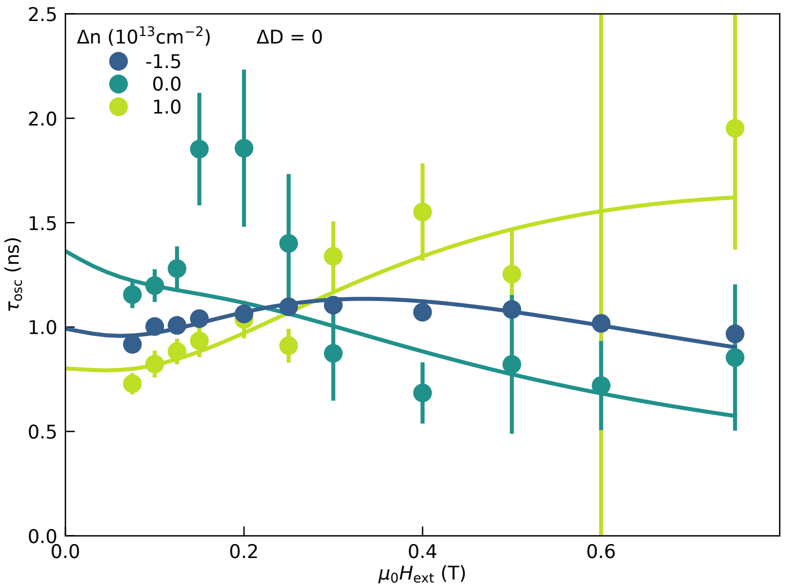

Fig. 7 Gate dependence of precession decay time. Gate dependence of the relation between decay time of the magnetization precession and H_{ext}. The circles and error bars indicate decay times with corresponding standard deviation extracted from the TRFE measurements. The solid lines correspond to the best fit of Eq. (9)

Δn (10^{13}cm^{-2})	α (10^{-3})	$\mu_0 \Delta H_{\text{int}}$ (mT)
-1.5	6.5 ± 0.3	9.0 ± 0.2
0	12 ± 3	5 ± 1
1.0	2 ± 1	12.6 ± 0.7

Table 1 Damping parameters. Fit results of the damping of the magnetization precession for three values of Δn with $\Delta D = 0$. The error in these values is the statistical error obtained from the least squares fitting procedure.

9 Detailed discussion on other possible mechanisms of the TRFE amplitude modulation caused by electrostatic gating

The change in amplitude of the oscillations in the TRFE signal with gating can have multiple causes. The most obvious one is a change in the magnetization precession

amplitude. It could however also be caused by a change in the strength of the Faraday effect. In this case, the TRFE curves for different values of the gate voltages (measured at the same external magnetic field) would only differ by scale factor, meaning that the curves can be mapped onto each other by simply scaling them along the vertical axis. This is clearly not happening in the measurements shown in Fig. 1a in the main text and Fig. 12, and therefore the change in amplitude is not caused by a pure change in the strength of the Faraday effect. Gate dependent magnetization curve measurements performed in the same setup, shown in Fig. 2, confirm that the strength of the Faraday effect is not significantly changed by electrostatic gating.

Another possibility would be that the equilibrium angle of $\mathbf{M}$ changes due to a change in the induced charge carrier density (Δn). This then would change the projection of the precession plane onto the propagation direction of the laser, and could change the TRFE amplitude without changing the magnetization precession amplitude. The canting would be due to a change in the effective internal field (H_{int}). However, our measurements show that the change in H_{eff} is less than 15%, which is not enough to explain the large increase in amplitude of the TRFE oscillations.

10 Complete data sets for the gate voltage dependence of the magnetization precession parameters

This section displays the complete data sets of the gate voltage dependence of the magnetization precession amplitude (A), starting phase (ϕ_0), and frequency (f).

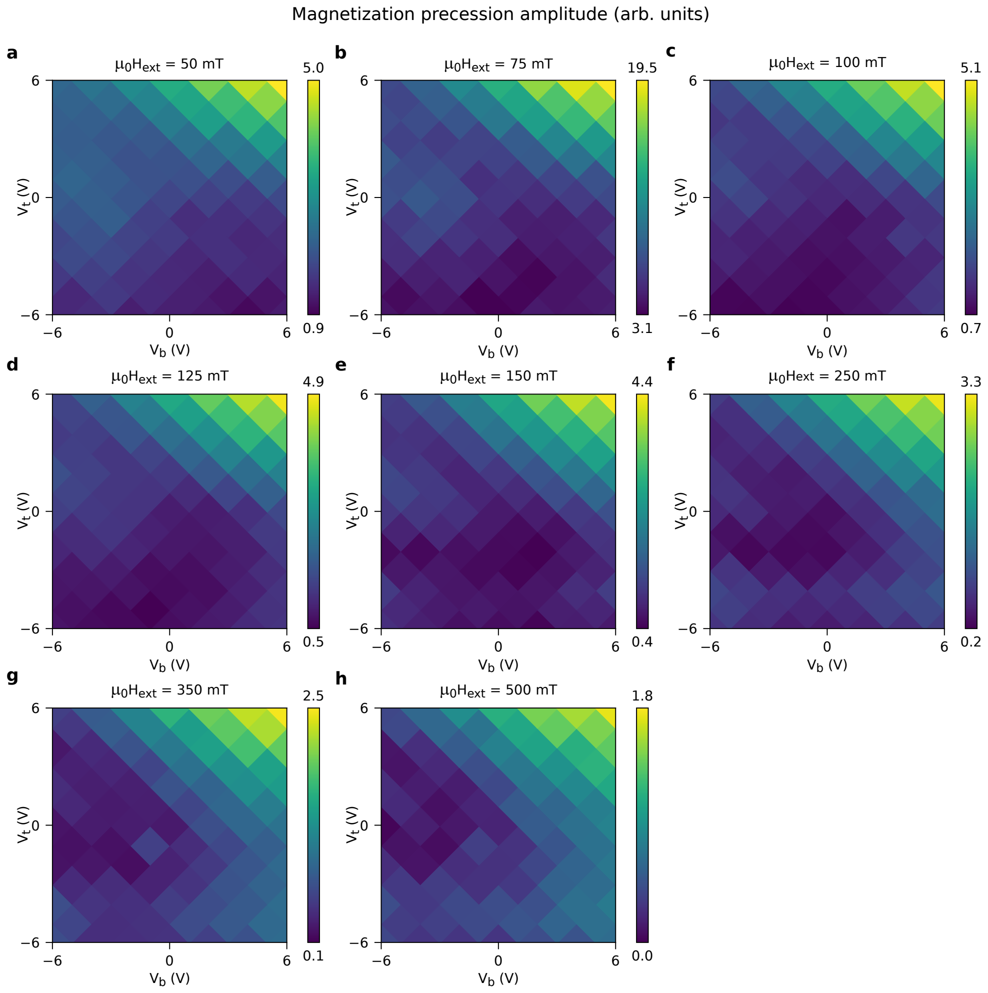

Fig. 8 Gate voltage dependence of the magnetization precession amplitude. a-h, The complete data set of the gate voltage dependence of the magnetization precession amplitude extracted from the TRFE measurements, for all values of the magnetic fields used during the measurements. The color scale has a different range for each plot. The scale bar limits indicate the minimum and maximum amplitude, all in the same arbitrary units, for $\mu_0 H_{\text{ext}}$ equal to 50 mT (**a**), 75 mT (**b**), 100 mT (**c**), 125 mT (**d**), 150 mT (**e**), 250 mT (**f**), 350 mT (**g**), and 500 mT (**h**).

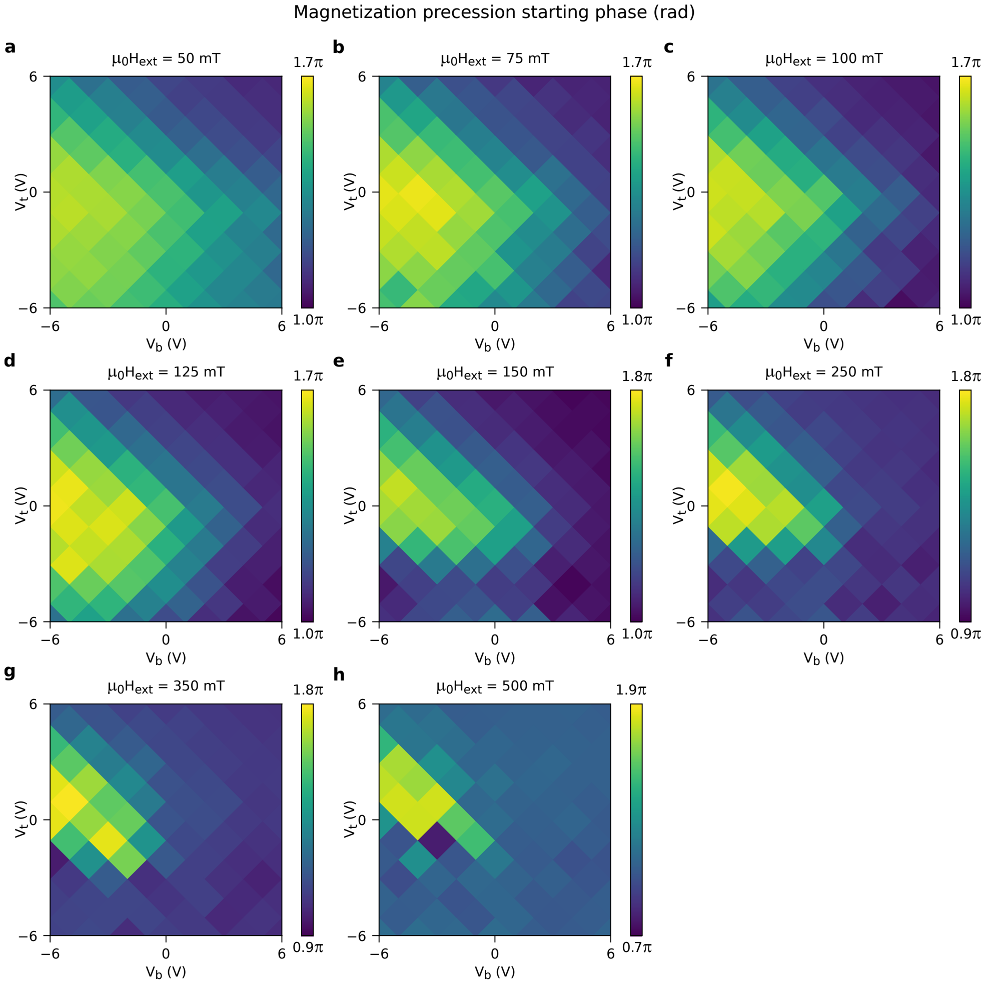

Fig. 9 Gate voltage dependence of the magnetization precession starting phase. a-h, The complete data set of the gate voltage dependence of the magnetization precession starting phase extracted from the TRFE measurements, for all values of the magnetic fields used during the measurements. The color scale has a different range for each plot. The limits indicate the minimum and maximum phase, in radians, for $\mu_0 H_{\text{ext}}$ equal to 50 mT (**a**), 75 mT (**b**), 100 mT (**c**), 125 mT (**d**), 150 mT (**e**), 250 mT (**f**), 350 mT (**g**), and 500 mT (**h**).

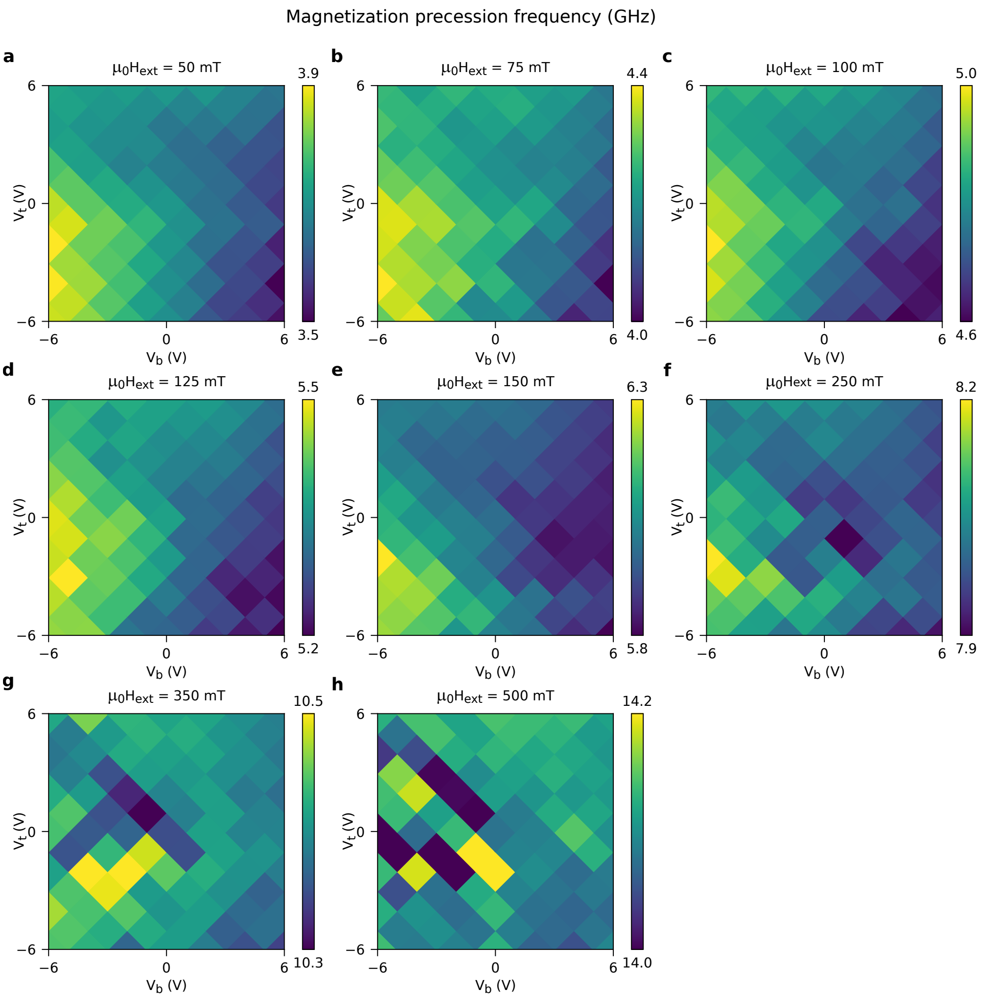

Fig. 10 Gate voltage dependence of the magnetization precession frequency for all magnetic fields. a-h, The complete data set of the gate voltage dependence of the magnetization precession frequency extracted from the TRFE measurements, for all values of the magnetic fields used during the measurements. The color scale has a different range for each plot. The limits indicate the minimum and maximum frequency, in GHz, for $\mu_0 H_{\text{ext}}$ equal to 50 mT (**a**), 75 mT (**b**), 100 mT (**c**), 125 mT (**d**), 150 mT (**e**), 250 mT (**f**), 350 mT (**g**), and 500 mT (**h**).

11 Magnetization precession frequency for negative magnetic fields

The measured magnetization precession frequency is found to be independent of the sign of H_{ext}, (Fig. 11). There are two outliers for $\Delta n = 0$ cm $^{-2}$ (at -0.75 T and -0.5 T), and one for $\Delta n = 1.0{\times}10^{-13}$ cm^{-2} (at -0.75 T). The precession amplitude in the TRFE measurements was too small for these measurements to obtain a good fit. Fig. 11b shows the difference in precession frequency for positive and negative fields, $f(H_{\text{ext}}) - f(-H_{\text{ext}})$. The absolute difference is smaller than 0.2 GHz, and typically smaller than 0.1 GHz (disregarding the outliers). Fig. 11c shows the relative difference in precession frequency for positive and negative fields, $2[f(H_{\text{ext}}) - f(-H_{\text{ext}})]/[f(H_{\text{ext}}) + f(-H_{\text{ext}})]$. Disregarding the outliers, the relative difference is smaller than 2%, and typically smaller than 1%. We point out that especially for $\Delta n = -1.5{\times}10^{13}$ cm^{-2}, for which the precession amplitude is very large, the frequency difference is very small (0.02 GHz, and 0.2% for fields above 125 mT). The TRFE traces at $\mu_0 H_{\text{ext}} = \pm 100$ mT are shown in Fig. 12. From these measurements it is already clear that reversing H_{ext} does not affect the frequency of the oscillations. These measurements also show again the arguments presented in the main text that the magnetization precession is not purely caused by the ΔK mechanism: both the amplitude and starting phase of the oscillations are different for positive and negative field. Furthermore, these measurements also show again the argument presented in the main text and Supplementary Section 9 that the gate dependence of the TRFE oscillation amplitude is not (purely) caused by a gate-induced change in the strength of the Faraday effect: the background on top of which the oscillations in the TRFE measurements are, scales differently with Δn than the amplitude of the oscillation.

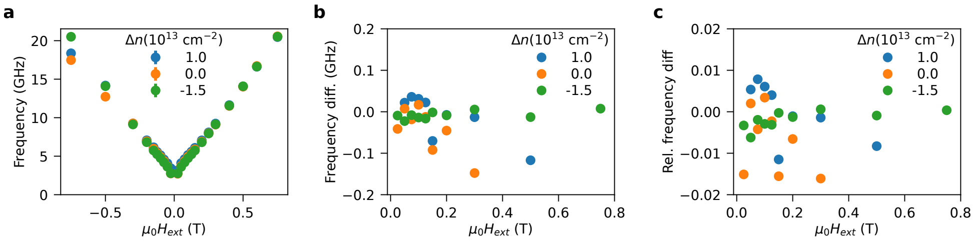

Fig. 11 H_{ext} **symmetry of the magnetization precession frequency**. **a**, Magnetization precession frequency for positive and negative external magnetic fields. **b**, Difference in precession frequency for positive and negative external magnetic fields. **c**, Relative difference in precession frequency for positive and negative external magnetic fields. All data presented has $\Delta D = 0$.

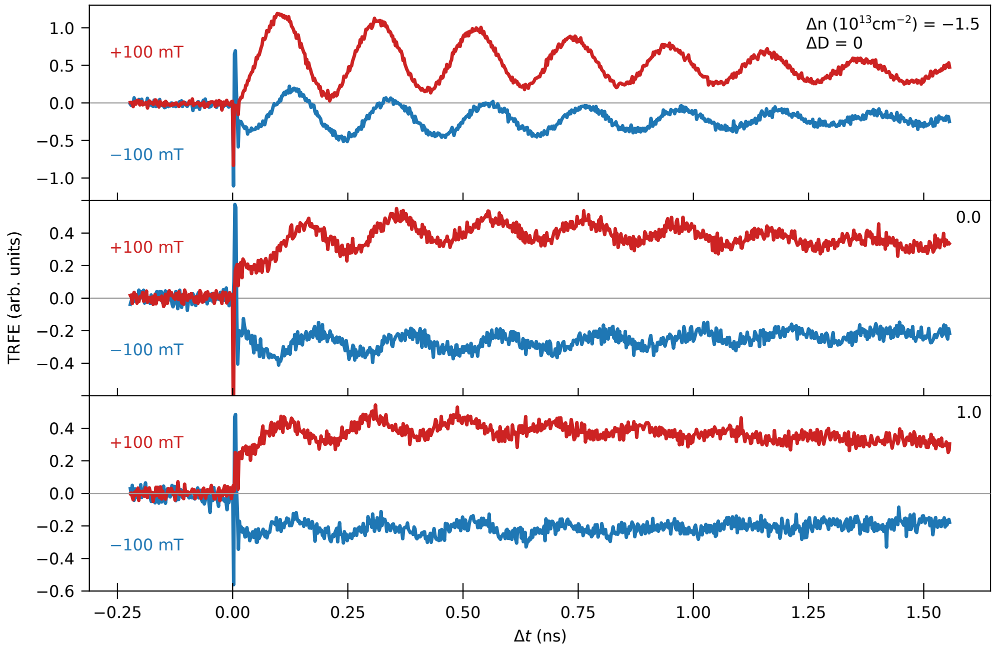

Fig. 12 TRFE measurements for positive and negative external magnetic fields. Time-resolved Faraday ellipticity measurements at positive and negative external magnetic field ($\mu_0 H_{\text{ext}} = \pm 100$ mT) and $\Delta D = 0$, for various values of Δn. The offset at $\Delta t < 0$ is set to zero for clarity. The difference in oscillation amplitude and starting phase for positive and negative H_{ext} is clearly visible. Note the different scale for the $\Delta n = -1.5 \times 10^{13}$ cm^{-2} data.

12 Detailed discussion on the contribution of non-linear magneto-optic effects to the H_{ext} asymmetry of the TRFE oscillations.

Our measurements show that the amplitude of the TRFE oscillations is not symmetric in the external magnetic field. This could be explained by a change in amplitude of the magnetization precession, as discussed in the main text. Another possibility is that the amplitude of the magnetization precession is still symmetric in H_{ext}, but the change in polarization of the probe by magneto-optic effects in the CGT is not. This could happen if the probe is not only affected by the Faraday effect, which is linear in the magnetization, but also by higher order magneto-optic (MO) effects, such as the Cotton-Mouton effect. The change in polarization of the probe (ψ) due to the MO effects of the sample depends on the sample magnetization as $\psi = a_i M_i + b_{ij} M_i M_j$, where the parameters a_i and b_{ij} are independent of the magnetization, $b_{ij} = b_{ji}$, and $i, j = x, y, z$. The Faraday effect is described by the first term, and the quadratic MO effect, e.g. Cotton-Mouton effect, by the second term. In the time-resolved Faraday ellipticity measurements, we observe variations in ψ caused by small variations in the

magnetization around the equilibrium magnetization M_0. This is expressed as:

$$\Delta\psi = a_i \Delta M_i + 2b_{ij} M_{0,i} \Delta M_j + b_{ij} \Delta M_i \Delta M_j. \tag{10}$$

We will now discuss how the quadratic MO effect could affect our TRFE measurements. The last term in Eq. (10) is quadratic in $\Delta\mathbf{M}$, and would result in a double frequency component in the TRFE oscillations. As this is not observed, this term is assumed to be negligible in our measurements, meaning that b_{ij} and therefore the second order MO effect is negligibly small, or that ΔM_i is much smaller than $M_{0,i}$

The second term in Eq. (10) is linear in $\Delta\mathbf{M}$, and could be of the same order of magnitude as the first term for arbitrarily small values of ΔM_i. If this is the case, i.e. if $b_{ij} M_{0,i}$ is of the order of a_i, this term will result in the oscillation amplitude of ψ being not symmetric in H_{ext}, even for a thermal induced precession where the oscillation amplitude of ΔM is symmetric in H_{ext}. This is because upon reversing H_{ext}, $M_{0,i}$ changes sign, changing ψ from $(a_j + b_{ij} M_{0,j})\Delta M_j$ to $(a_j - b_{ij} M_{0,j})\Delta M_j$. Therefore, a quadratic MO effect could result in the observed asymmetry in the amplitude of the TRFE oscillations while the amplitude of the magnetization precession is still symmetric in H_{ext}.

However, even though quadratic MO effects could explain that the TRFE oscillation amplitude is asymmetric in H_{ext} for a purely thermal excitation of the magnetization precession, they cannot explain all of our observations. For example, the pump fluence dependence of the TRFE measurements presented in Fig. 5e shows a jump in the signal at $\Delta t = 0$ of which the sign depends on the pump fluence. For convenience, the data is presented again in Fig. 13a, with a close-up of the interesting part in Fig. 13b. The data shows that for high pump fluences, the TRFE signal increases sharply right after $\Delta t = 0$, indicating a fast decrease in, or canting of, the magnetization. For the lowest pump fluence however, the signal sharply *decreases* right after $\Delta t = 0$. The argument for why a purely thermal excitation cannot explain this, as detailed below.

For a purely thermal demagnetization, the magnetization and the magnetocrystalline anisotropy decrease right after excitation [6]. Canting of the magnetization only happens if the magnetocrystalline anisotropy briefly changes on a time scale shorter than the precession period. This is then accompanied by a starting phase that is close to $\pm\pi/2$, i.e. if the oscillations start as a sine instead of a cosine (in our experimental geometry) [7]. Our measurements however show a clear cosine-like start of the oscillations. Therefore the magnetization only decreases during the heating by the laser. Assuming the direction of the magnetization, $M_i/|M|$, has not changed during the excitation, the TRFE signal can be expressed in terms of the magnitude of the magnetization. Doing this in Eq. (10) yields:

$$\psi = a_i M_i + b_{ij} M_i M_j = A|M| + B|M|^2 \tag{11}$$

$$\Delta\psi = A\Delta|M| + 2B|M_0|\Delta|M| = (A + 2B|M_0|)\,\Delta M. \tag{12}$$

where $A = a_i M_i/|M|$ and $B = b_{ij} M_i M_j/|M|^2$ are constant. The sign of $\Delta\psi$ is thus independent of ΔM and therefore independent of the fluence of the pump. Hence the

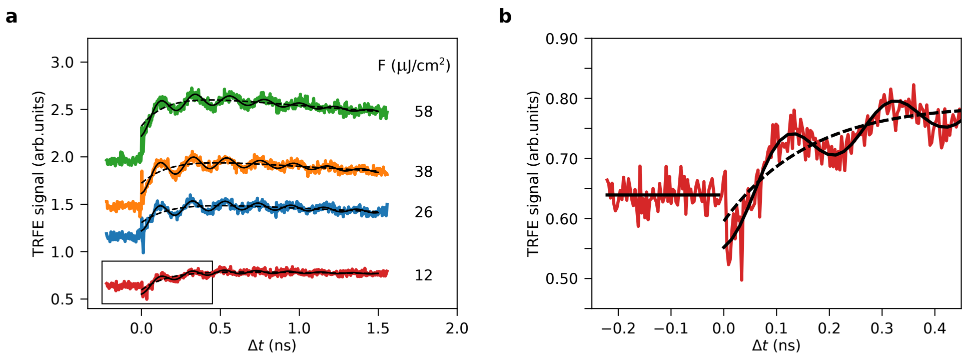

Fig. 13 Supporting evidence for coherent optical excitation of the magnetization precession. a, Pump fluence dependence of the TRFE measurements for $V_t = -6V$, $V_b = 6V$ ($D = 1.1$ V/nm, $\Delta n = 0$) and $\mu_0 H_{\text{ext}} = 100$ mT. The solid line is the best fit of the data, using Eq. (2) of the Methods for $\Delta t > 0$, and a constant for $\Delta t < 0$. The dashed lines indicate the double exponential background of the oscillation **b**, Close-up of the data for a pump fluence of 12 μJ/cm^2, indicated by the black rectangle in **a**.

change from a sharp increase to a sharp decrease by changing the power of the pump cannot be explained by a pure thermal excitation process.

A coherent excitation on the other hand, caused by e.g. the inverse Cotton-Mouton effect, does have the ability to create an effective magnetic field to cant the magnetization on sub-picosecond timescales [8]. The combination of thermal excitation and coherent laser excitation could explain the observed behavior of the TRFE with pump power. How this combination can explain the asymmetry in the amplitude and phase with external magnetic field is explained in Supplementary section 13 below.

13 Model for optical excitation of magnetization dynamics

In this section we develop a model for the excitation of the magnetization dynamics, including coherent optical excitations, and thermal effects. Using this model, we obtain the amplitude and starting phase of the magnetization precession.

The coherent effects we consider are the inverse Cotton-Mouton effect (ICME) and photo-induced magnetic anisotropy (PIMA). The effective magnetic field generated by these effects is described by [9]:

$$H_{\text{eff},i} = G_{ijkl} M_i E_k E_l \tag{13}$$

where G_{ijkl} is a rank 4 polar tensor that is symmetric under exchanging the first or last pair of indices, M_i the magnetization, E_i the electric field of the light, and $i, j, k, l = x, y, z$. Note that the two effects are described by two different tensors. The crystal symmetry of the CGT (space group $R\bar{3}$ [10]) restricts the number of

independent elements of these tensors to 12, and forces some to be zero, as indicated in Tab. 2. In this table, G_{ij} is an abbreviation of G_{klmn}, where i and j take the values 1 to 6, determined by kl and mn respectively. The values for i (j) = 1, 2 and 3 correspond to kl $(mn) = xx$, yy and zz, while the values 4, 5 and 6 correspond to kl (mn) consisting of the combination of y and z, x and z, and x and y respectively. The 4 boxed elements are responsible for generating an effective magnetic field in the y direction when both the magnetization and the polarization of the pump are in the xz plane. These elements are needed to explain the observed asymmetry of the amplitude in the TRFE oscillations for positive and negative values of H_{ext}.

G_{ij}		j = 1	2	3	4	5	6
	1	G_{11}	G_{12}	G_{13}	G_{14}	G_{15}	G_{16}
	2	G_{12}	G_{11}	G_{13}	$-G_{14}$	$-G_{15}$	$-G_{16}$
	3	G_{31}	G_{31}	G_{33}	0	0	0
i	4	$\boxed{G_{41}}$	$-G_{41}$	0	G_{44}	$\boxed{-G_{54}}$	$-G_{51}$
	5	G_{51}	$-G_{51}$	0	G_{45}	G_{44}	G_{41}
	6	$\boxed{-G_{16}}$	G_{16}	0	$-G_{15}$	$\boxed{G_{14}}$	$(G_{11}-G_{12})/2$

Table 2 Symmetry-adapted form of $\mathbf{G_{ijkl}}$. This table, calculated using Ref. [11], shows the relation between the different elements of G_{ijkl} imposed by the space group $R\bar{3}$ and the invariance under interchanging i and j, and k and l. using The 12 independent elements are indicated in black, the dependent ones in gray. The elements that are zero are highlighted in red. The boxed elements are responsible for generating an effective magnetic field in the y direction when both the magnetization and the polarization of the pump are in the xz plane. Conversion between G_{klmn} and G_{ij} is explained in the text.

To determine the effect of the ICME and photo-induced magnetic anisotropy on the precession, we use the following simplified model. Light travels in the direction $\widehat{\mathbf{k}} = (\sin\theta_k, 0, \cos\theta_k)$, where θ_k the angle between $\widehat{k}$ and the z-axis. The orientation of the axes is indicated in Fig. 3e of the main text. The polarization of the pump is linear, and in the xz plane. Its electric field vector is described by $\mathbf{E} = E_0(\cos\theta_k, 0, \sin\theta_k)$, where E_0 is the amplitude of the electric field. In equilibrium, the magnetization lies in the xz plane too: $\mathbf{M} = M_s(\sin\theta_M, 0, \cos\theta_M)$. Plugging the above expression in Eq. (13) reveals that the components of effective magnetic field induced by these coherent optical excitations depends sinusoidally on θ_M and on $2\theta_k$, where the amplitude and phase are in general different for the different components of $H_{\text{eff},i}$.

When the pump pulse hits the sample, we assume that the magnetization is firstly affected by the ICME. This effect can be described by a delta pulse in the effective magnetic field, resulting in an instantaneous rotation of the magnetization [8, 9, 12]. Afterwards, the effective magnetic field is changed instantaneously to a new value due to both the PIMA and a thermal induced change in the magnetocrystalline anisotropy. The former changes the effective magnetic field according to Eq. (13). The latter decreases the magnetocrystalline anisotropy of the sample, resulting in a decrease of the effective internal field along the sample normal [6]. From this point on, the

magnetization starts to precess in a circular orbit around the effective magnetic field, as described by the Landau-Lifshitz-Gilbert equation.

The time dependence of $\mathbf{M}$ for negligible damping is conveniently given by Rodrigues' rotation formula:

$$\mathbf{M}(t) = \mathbf{M}_0 \cos \omega t + \left(\widehat{\mathbf{H}}_{\text{eff}} \times \mathbf{M}_0\right) \sin \omega t + \widehat{\mathbf{H}}_{\text{eff}} \left(\widehat{\mathbf{H}}_{\text{eff}} \cdot \mathbf{M}_0\right) (1 - \cos \omega t) \tag{14}$$

which rotates the vector $\mathbf{M}_0$ around a unit vector $\widehat{\mathbf{H}}_{\text{eff}}$ with angular frequency ω.

The Faraday effect is assumed to be only sensitive to changes in the magnetization along the propagation direction of the laser, $\widehat{\mathbf{k}}$. The amplitude and phase of the precession as measured by Faraday ellipticity are therefore obtained by projecting the motion of the magnetization on the propagation direction of the laser. This is achieved by taking the inner product of $\mathbf{M}(t)$ and $\widehat{\mathbf{k}}$. The resulting expression has a term with time dependence $\cos(\omega t)$, and a term with time dependence $\sin(\omega t)$. By combining the two into a single phase shifted cosine, the amplitude and starting phase of the TRFE oscillations are obtained. The amplitude angle (θ_A) of the precession, which is the angle between the magnetization and the effective magnetic field, is calculated too.

For the calculations, we use the experimental parameters $\mu_0 \mathbf{H}_{\text{int}} = 125$ mT and $\theta_H = 50$ degrees. We set the small thermal induced change of the effective field $\mu_0 \Delta H_{\text{thermal}}$, caused by a reduction of the magnetocrystalline anisotropy and the magnetization, to -1 mT. A good qualitative agreement with the TRFE oscillation amplitude, and a good quantitative agreement with the starting phase phase, shown in Fig. 3c,d of the main text, are obtained if the ICME and the PIMA depend on the orientation of the magnetization as $\sin(\theta_M - 50°)$. The ICME then rotates $\mathbf{M}$ along a fixed direction ($\widehat{\mathbf{H}}_{\text{ICME}}$), and the PIMA generates an effective field H_{PIMA} along a fixed axis, of which the orientations are independent of the value of H_{ext}. Furthermore, for the angle over which the ICME rotates M (θ_{ICME}), we took $0.4° \sin(\theta_M - 50°)$ along the vector $\widehat{\mathbf{H}}_{\text{ICME}} = (-0.19, 0.96, 0.19)$, and $\mu_0 \Delta H_{\text{PIMA}} = 0.4 \sin(\theta_M - 50°)$ mT along the direction $(0.19, 0.96, -0.19)$.

The resulting precession angle, and the TRFE oscillation amplitude and starting phase are shown in Fig. 14. The values are only plotted for external field magnitudes larger than 75 mT, since at lower values the magnetization is not fully saturated after laser excitation.

The external magnetic field dependence of both θ_{A} and the Faraday ellipticity amplitude are not symmetric in H_{ext}, and have an inflection point around $\mu_0 H_{\text{ext}} = \pm 200$ mT. This agrees with the measured amplitude for $\Delta n = -1.5 \times 10^{13}$ cm^{-2} and 1.0×10^{13} cm^{-2}. A possible reason for the discrepancy for $\Delta n = 0$ is given below. The rate at which the measured amplitude decreases for large fields, and the asymmetry in this rate, is much better captured by θ_A than by the calculated Faraday ellipticity amplitude. This could mean that the calculation of the TRFE signal from the magnetization precession is too simplistic in our model model.

The calculated starting phase (ϕ_0) also captures the features of the measured starting phase. For positive fields, ϕ_0 is mostly constant, at a value slightly below π, and starts to increase as the field approaches zero. At negative fields, the phase increases as the magnitude of the field grows, saturating to a value of about 1.7π. This

is in quantitative agreement with our measurements for $\Delta n = -1.5 \times 10^{13}$ cm^{-2} and 1.0×10^{13} cm^{-2}, and in qualitative agreement for $\Delta n = 0$.

The behavior for $\Delta n = 0$ is very different from the other two. The amplitude is mostly symmetric in H_{ext} and the relative change with H_{ext} for low field is much faster. Also, the phase appears to be shifted to more positive values of H_{ext}. A possible explanation for this is that the thermally induced change in H_{eff} does not happen instantaneously, but on a time scale close to the period of the magnetization precession for low fields. In that case, the amplitude and phase can both be greatly affected if the two time scales are close. The strengths of the coherent excitations can also be different than for $\Delta n = 1.0$ or -1.5×10^{13} cm^{-2}.

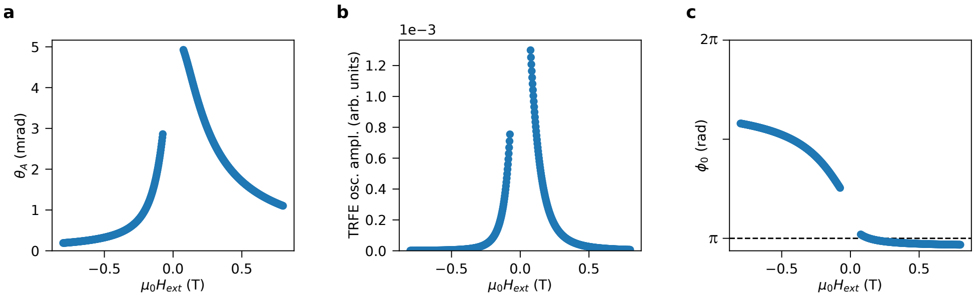

Fig. 14 Model for the effect of coherent excitations on the magnetization precession. a, The angle between the magnetization and the effective magnetic field versus H_{ext}. **b**, the amplitude of the percession versus H_{ext}, as measured by Faraday ellipticity, **c**, The starting phase of the precession versus H_{ext}.

References

[1] Lohmann, M., Su, T., Niu, B., Hou, Y., Alghamdi, M., Aldosary, M., Xing, W., Zhong, J., Jia, S., Han, W., Wu, R., Cui, Y.-T., Shi, J.: Probing Magnetism in Insulating Cr2Ge2Te6 by Induced Anomalous Hall Effect in Pt. Nano Letters **19**, 2397–2403 (2019)

[2] Mizukami, S., Sajitha, E.P., Watanabe, D., Wu, F., Miyazaki, T., Naganuma, H., Oogane, M., Ando, Y.: Gilbert damping in perpendicularly magnetized Pt/Co/Pt films investigated by all-optical pump-probe technique. Applied Physics Letters **96** (2010)

[3] Fang, Y., Wu, S., Zhu, Z.-Z., Guo, G.-Y.: Large magneto-optical effects and magnetic anisotropy energy in two-dimensional Cr2Ge2Te6. Physical Review B **98**, 125416 (2018)

[4] Zhang, T., Chen, Y., Li, Y., Guo, Z., Wang, Z., Han, Z., He, W., Zhang, J.: Laser-induced magnetization dynamics in a van der Waals ferromagnetic Cr2Ge2Te6 nanoflake. Applied Physics Letters **116**, 223103 (2020)

[5] Zollitsch, C.W., Khan, S., Nam, V.T.T., Verzhbitskiy, I.A., Sagkovits, D., O'Sullivan, J., Kennedy, O.W., Strungaru, M., Santos, E.J.G., Morton, J.J.L., Eda, G., Kurebayashi, H.: Probing spin dynamics of ultra-thin van der Waals magnets via photon-magnon coupling. Nature Communications **14**, 2619 (2023)

[6] van Kampen, M., Jozsa, C., Kohlhepp, J.T., LeClair, P., Lagae, L., de Jonge, W.J.M., Koopmans, B.: All-optical probe of coherent spin waves. Physical Review Letters **88**, 227201 (2002)

[7] Dalla Longa, F.: Laser-induced magnetization dynamics. PhD thesis, Eindhoven University of Technology (2008)

[8] Kalashnikova, A.M., Kimel, A.V., Pisarev, R.V., Gridnev, V.N., Usachev, P.A., Kirilyuk, A., Rasing, T.: Impulsive excitation of coherent magnons and phonons by subpicosecond laser pulses in the weak ferromagnet FeBO3. Physical Review B **78**, 104301 (2008)

[9] Yoshimine, I., Satoh, T., Iida, R., Stupakiewicz, A., Maziewski, A., Shimura, T.: Phase-controllable spin wave generation in iron garnet by linearly polarized light pulses. Journal of Applied Physics **116**, 043907 (2014)

[10] Carteaux, V., Brunet, D., Ouvrard, G., Andre, G.: Crystallographic, magnetic and electronic structures of a new layered ferromagnetic compound Cr2Ge2Te6. Journal of Physics: Condensed Matter **7**, 69–87 (1995)

[11] Gallego, S.V., Etxebarria, J., Elcoro, L., Tasci, E.S., Perez-Mato, J.M.: Automatic calculation of symmetry-adapted tensors in magnetic and non-magnetic materials: a new tool of the Bilbao Crystallographic Server. Acta Crystallographica Section A Foundations and Advances **75**, 438–447 (2019)

[12] Shen, L.Q., Zhou, L.F., Shi, J.Y., Tang, M., Zheng, Z., Wu, D., Zhou, S.M., Chen, L.Y., Zhao, H.B.: Dominant role of inverse Cotton-Mouton effect in ultrafast stimulation of magnetization precession in undoped yttrium iron garnet films by 400-nm laser pulses. Physical Review B **97**, 224430 (2018)
